# Females with angina pectoris have altered lipoprotein metabolism with elevated cholesteryl ester transfer protein activity and impaired high-density lipoproteins-associated antioxidant enzymes

**DOI:** 10.3892/ijmm.2011.874

**Published:** 2011-12-29

**Authors:** JUNGHO PARK, JAE-RYONG KIM, DONG-GU SHIN, KYUNG-HYUN CHO

**Affiliations:** 1 School of Biotechnology, Yeungnam University, Gyeongsan 712-749; 2 Department of Biochemistry and Molecular Biology, Aging-associated Vascular Disease Research Center, Yeungnam University, Daegu 705-717; 3 Cardiovascular Division, Internal Medicine, Aging-associated Vascular Disease Research Center, Yeungnam University Medical Center, Daegu 705-717; 4 Research Institute of Protein Sensor, Yeungnam University, Gyeongsan 712-749, Republic of Korea

**Keywords:** angina pectoris, lipoproteins, apolipoproteins, cholesteryl ester transfer protein

## Abstract

In order to investigate non-invasive biomarkers for angina pectoris (AP), we analyzed the lipid and protein composition in individual lipoproteins from females with angina pectoris (n=22) and age- and gender-matched controls (n=20). In the low-density lipoprotein (LDL) fraction, the triglycerides (TG) and protein content increased in the AP group compared to the control group. The AP group had lower total cholesterol (TC) and elevated TG in the high-density lipoprotein (HDL) fraction. In the AP group, cholesteryl ester transfer protein (CETP) activity was enhanced in HDL and LDL, while lecithin:cholesterol acyltransferase (LCAT) activity in HDL_3_ was almost depleted. Antioxidant activity was significantly decreased in the HDL_3_ fraction, with a decrease in the HDL_2_ particle size. In the HDL_3_ fraction, paraoxonase and platelet activating factor-acetylhydrolase (PAF-AH) activity were much lower and the levels of CETP and apoC-III were elevated in the AP group. The LDL from the AP group was more sensitive to cupric ion-mediated oxidation with faster mobility. In conclusion, the lipoprotein fractions in the AP group had impaired antioxidant activity and increased TG and apoC-III with structural and functional changes.

## Introduction

Coronary artery syndrome is the most common cause of death among women in developed countries (1). The coronary artery syndrome is initiated by atherosclerosis, which is complexed with dyslipidemia and an inflammatory process.

Angina pectoris (AP) is paroxysmal thoracic pain that is sometimes accompanied by a feeling of suffocation (2). AP is most often due to ischemia of the myocardium and is precipitated by effort or excitement. Several biomarkers have been developed to diagnose coronary artery disease, including lipid and inflammatory markers (3), although the markers are not prognostic. It is well-known that the apoB/apoA-I ratio is important to predict the risk of coronary artery disease (CAD) (4). There have been many non-invasive biochemical measures used to predict cardiovascular risk, such as lipid and lipoprotein metabolism, inflammation, and oxidative stress (5–7). Recently, the apolipoprotein composition in lipoprotein and high-density lipoprotein (HDL) sub-fractions has been shown to change in the sera from patients with acute coronary syndrome (ACS). Huang *et al* (8) reported that plasma ApoAV is associated with ACS. Tashiro *et al* (9) reported that pre β1-HDL is elevated in patients with unstable angina pectoris. Furthermore, we recently reported an increase in apoC-III in HDL_2_ from a male with a myocardial infarction (10). Similarly, Lee *et al* (11) reported that low-density lipoprotein (LDL)-containing apoC-III is an independent risk factor for coronary events in diabetic patients. These findings collectively raised the possibility of a relationship between increased lipid and apoC-III, oxidative modification, and inflammatory processes. In ACS, oxidative stress constitutes an integral part of plaque rupture and platelet activation (12). The oxidative modification of LDL, which is considered a strong risk factor for atherosclerosis and ACS, occurs through the release of pro-inflammatory and oxidative signals. The composition and functional correlations of HDL is also associated with the incidence of metabolic syndrome as described in our previous report (13). Elevated triglycerides (TG) and low cholesterol (C) content in HDL is a major characteristic of the metabolic syndrome (14) and of myocardial infarction (MI) (10). A low HDL-C level is the most common lipid abnormality observed in families with premature coronary heart disease (CHD) (15).

There have been many studies attempting to establish non-invasive biomarkers for the early detection of risk for CHD, including AP and MI. In the current study, to detect unique parameters in lipoprotein levels, lipid and apolipoprotein compositions, and enzyme activities were analyzed between females with AP and controls.

## Materials and methods

### Patients and controls

Female patients with stable AP (n=22) were selected using the following criteria: the presence of chest or arm discomfort that is rarely described as pain, but is reproducibly associated with physical exertion or stress and relieved within 5–10 min of rest and/or administration of sublingual nitroglycerin. The diagnosis was confirmed with a treadmill exercise test and coronary angiography in all patients. Patients did not take any medications, except for statins, prior to hospitalization. Age- and gender-matched reference subjects (n=20) were recruited from healthy volunteers who underwent regular health evaluations at the Health Center of Yeungnam University Hospital (Daegu, Korea). They had unremarkable medical records without a history of endocrinological disorders. Heavy alcohol consumers (>30 g EtOH/day) and those who had taken prescribed drugs to treat hyperlipidemia, diabetes mellitus, or hypertension were excluded. Informed consent was obtained from all patients and the control group prior to enrollment in the study. The Institutional Review Board at the Medical Center of Yeungnam University approved the protocol.

### Isolation of lipoproteins

After overnight fasting, blood was collected using a vacutainer (BD Bio Sciences, Franklin Lakes, NJ, USA) containing EDTA (final concentration, 1 mM). Plasma was isolated by low-speed centrifugation and stored at −80˚C until analysis.

Very low-density lipoproteins (VLDL, d<1.019 g/ml), LDL (1.019<d<1.063), HDL_2_ (1.063<d<1.125) and HDL_3_ (1.125<d<1.225) were isolated from individual patient and control sera via sequential ultracentrifugation (16), with the density adjusted by the addition of NaCl and NaBr in accordance with standard protocols. Samples were centrifuged for 24 h at 10˚C at 100,000 × g using a Himac CP 90α (Hitachi, Tokyo, Japan) at the Instrumental Analysis Center of Yeungnam University.

For each of the lipoproteins which were individually purified, total cholesterol (TC) and TG measurements were obtained using commercially available kits (cholesterol, T-CHO, and TG, Cleantech TS-S; Wako Pure Chemical, Osaka, Japan). The protein concentrations of lipoproteins were determined via the Lowry protein assay, as modified by Markwell *et al* (17) using the Bradford assay reagent (Bio-Rad, Seoul, South Korea) with bovine serum albumin (BSA) as a standard. To assess the degree of oxidation of individual LDL, the concentration of oxidized species in LDL was determined by the thiobarbituric acid reactive substances (TBARS) method using malondialdehyde (MDA) as a standard (18).

### Ferric reducing ability of plasma assay

The ferric reducing ability of plasma (FRAP) was determined using the method described by Benzie and Strain (19) with a slight modification, as described previously (20). The antioxidant activities of the individual HDL fractions (20 μg each) were then estimated by measuring the increase in absorbance induced by the generated ferrous ions.

### Cholesteryl ester conversion assay

Cholesteryl ester conversion was performed via lecithin:cholesterol acyltransferase (LCAT) assays, as previously described (21). An equal amount of individual lipoproteins (in 50 μl) from each patient was utilized as the enzyme source. ApoA-I-rHDL containing radiolabeled cholesterol (1 μCi of [^14^C]-4-cholesterol/69 μg of cholesterol/1 mg of apoA-I) was used as a substrate, and the apoA-I was then expressed using an *E. coli* expression system, as described previously (21). Discoidal rHDL was prepared via the sodium cholate dialysis method using initial molar ratios of palmitoyloleoyl phosphatidylcholine (POPC)-cholesterol-apoA-I-sodium cholate at a ratio of 95:5:1:150 (wt/wt/wt/wt). The reaction was initiated by the addition of individual serum, and the mixture was then incubated for 1 h at 37˚C. Next, the esterified cholesterol and free cholesterol were separated via thin layer chromatography, and the activity was expressed as the percentage conversion rate of cholesteryl ester from free cholesterol.

### Cholesteryl ester transfer assay

An rHDL-containing apoA-I and cholesteryl oleate was synthesized in accordance with the method described by Cho (20) using trace amounts of [^3^H]-cholesteryl oleate (TRK886, 3.5 μCi/mg of apoA-I; GE Healthcare) with a slight modification (22). The CE-transfer reaction was allowed in 300 μl reaction mixtures that contained equal amounts of the individual lipoproteins (20 μl, 10–20 μg of protein) as a cholesteryl ester transfer protein (CETP) source and rHDL-agarose (50 μl, 0.25 mg/ml) and human LDL (50 μl, 0.25 mg/ml) as a CE-donor and CE-acceptor, respectively. After incubation at 37˚C, the reaction was halted via brief centrifugation (10,000 × g) for 3 min at 4˚C. The supernatant (150 μl) was then subjected to scintillation counting, and the percentage transfer of [^3^H]-CE from rHDL to LDL was calculated.

### Paraoxonase assay

Paraoxonase-1 (PON-1) activity toward paraoxon was determined by evaluating the hydrolysis of paraoxon into *p*-nitrophenol and diethylphosphate, which was catalyzed by the enzyme (23). PON-1 activity was then determined by measuring the initial velocity of *p*-nitrophenol production at 37˚C, as determined by measuring the absorbance at 405 nm (microplate reader, Bio-Rad model 680; Bio-Rad, Hercules, CA, USA), as described previously (13).

### Platelet activating factor-acetylhydrolase (PAF-AH) assay

The individual lipoprotein fractions (10 μl, 20 μg) were used as an enzyme source for the PAF-AH reaction with an Lp-PLA_2_ assay conducted according to the method described by Boyd *et al* (24). Briefly, [^3^H]-platelet activating factor (hexadecyl-2-acetyl sn-glyceryl-3-phosphorylcholine, NET910, 0.1 mCi/ml; Perkin-Elmer Life and Analytical Sciences, Boston, MA, USA) and 1-O-hexadecyl-2-acetyl-sn-glycero-3-phosphocholine were used as substrates for the reaction. A substrate solution containing 10 μl of [^3^H]-PAF (1 μCi, 50 μM) and 12 μM of cold PAF was incubated using each HDL solution as a source for 30 min. The reaction was then stopped by vortexing the solutions with 600 μl of CHCl_3_:MeOH (2:1, v/v), after which the aqueous layer (150 μl) was removed. The aqueous layer was then vortexed again with CHCl_3_, after which it was centrifuged and the upper phase was used for scintillation counting.

### Electromobility of lipoproteins

In order to compare the electromobility of the patient and control samples, the migration of each lipoprotein (LDL, HDL_2_ and HDL_3_) was evaluated by agarose electrophoresis. The gels were then dried and stained with 0.125% Coomassie Brilliant Blue, after which the relative band intensities were compared via band scanning using Gel Doc^®^ XR (Bio-Rad) with Quantity One software (version 4.5.2).

### Western blot analysis

The apolipoprotein/lipoprotein compositions were compared via sodium dodecyl sulfate-polyacylamide gel electrophoresis (SDS-PAGE) with identical protein loading quantities (6 μg of total protein per lane) from individual HDL_3_, and the levels of expression of apolipoprotein were analyzed via immunodetection. Anti-human apoC-III antibody (AB821) was purchased from Chemicon (Temecula, CA, USA). Anti-human CETP antibody (ab19012) and LCAT antibody (ab786) were purchased from Abcam (Cambridge, UK). The relative band intensity (BI) was compared via band scanning with Gel Doc^®^ XR (Bio-Rad) using the Quantity One software (version 4.5.2).

### Data analysis

All data are expressed as the mean ± SD from at least three independent experiments with duplicate samples. Data comparisons were assessed by the Student’s t-test using the SPSS program (version 14.0; SPSS, Inc., Chicago, IL, USA).

## Results

### Lipid and protein composition in lipoprotein

The serum TC concentrations were similar between the groups (204±57 and 200±32 mg/dl, respectively), which remained in the normal range, as suggested by the guidelines of the National Cholesterol Education Program (NCEP)-adult treatment panel (ATP)-III. The LDL-C level was similar between the groups (105±38 and 108±33 mg/dl for the AP patients and controls, respectively). However, the HDL-C level was slightly lower in the AP patients than the controls. The ratio of HDL-C-to-TC was significantly lower in the AP patients (34±2%) compared to the control group (40±4%). The serum TG level was not significantly different between the groups (136–175 mg/dl).

**Figure 1:**
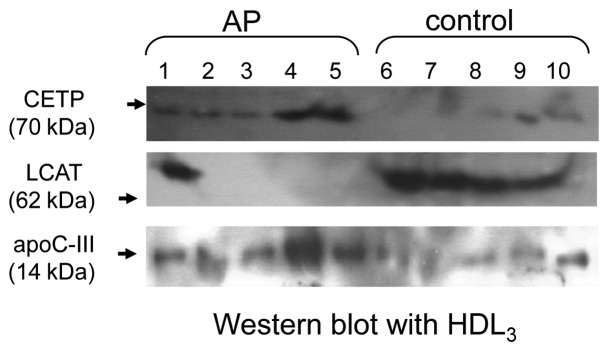
Immunodetection of apolipoproteins and enzymes in HDL_3_ between the angina pectoris (AP) and control groups. Equal amounts of HDL_3_ (6 μg of protein) from an individual subject were loaded per lane. Polyclonal CETP antibody (Abcam, ab19012), apoC-III antibody (Chemicon, AB821), and LCAT antibody (Abcam, ab786) were used as primary antibodies.

Properties of lipoprotein are good biomarkers which reflect the progress of cardiovascular and renal disease. As shown in Table I, the AP group had a similar composition of lipid and protein in VLDL with the control group. Although the TC and protein content in LDL was similar between the groups, the TG content in LDL was significantly higher in the AP group (38 mg of TG/mg of TP) compared to the control group (30 mg of TG/mg of TP). In the HDL_2_ fraction, the AP group had a much lower TC content and a higher TG content (44% higher TC and 32% lower TG) than the control group. Immunodetection revealed that the level of expression of apoC-III was elevated in the HDL_3_ fraction of the AP group (Fig. 1).

### CETP and LCAT activity

As shown in Table II, although the CE-transfer activity of the VLDL fraction was similar between the groups (~2–3% CE-transfer), the LDL fraction of the AP group had 2-fold increased CE-transfer activity. The CETP activity of the HDL fraction also increased in the AP group (a 70 and 34% increase for HDL_2_ and HDL_3_, respectively), compared to the control. Immunodetection revealed that CETP was highly expressed in the HDL_3_ fraction of the AP group (Fig. 1).

The LCAT activity was significantly lower in the HDL_3_ fraction of the AP group, while no difference existed in the HDL_2_ fraction between the groups. The LCAT activity for CE-conversion from FC was lowered in the HDL_2_ fraction in the AP and control groups, as shown in Table II. The level of expression of LCAT was nearly undetectable in the AP group (lane 1–5) except in one patient (Fig. 1).

### Antioxidant activity of HDL_2_ and HDL_3_ was decreased in the AP group

**Figure 2:**
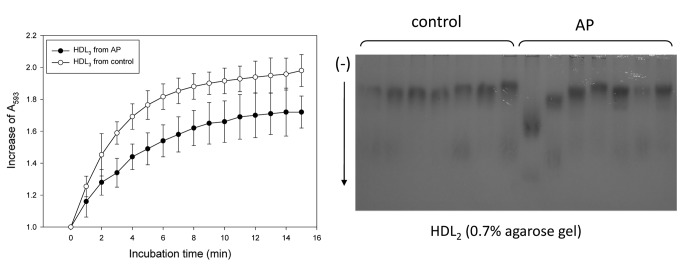
Comparison of the antioxidant activity of HDL. Reduction potential of HDL_3_ based on the ferric-reducing ability of plasma (FRAP). The same amount of HDL_3_ (0.05 ml, 2 mg/ml) was added to the substrate solution. Comparison of electrophoretic mobility of HDL_2_ on 0.7% agarose gels without cupric ion treatment.

**Figure 3:**
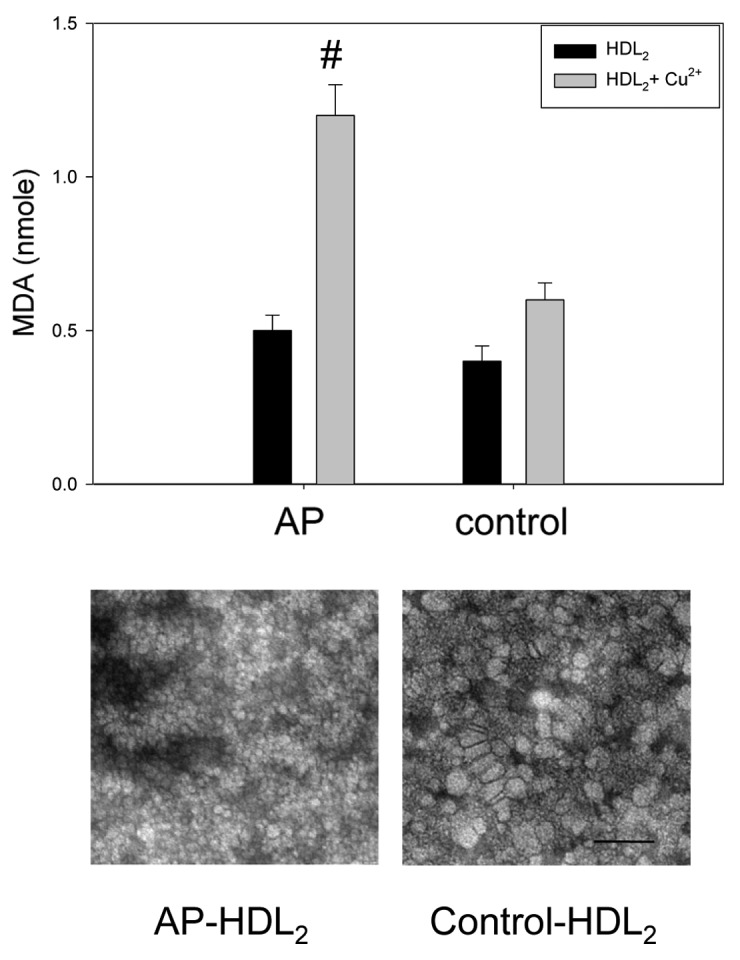
Properties of HDL_2_ from the AP and control groups. Susceptibility to oxidation in HDL_2_ by cupric ion treatment (graph). Representative picture of negatively-stained HDL_2_ from the angina pectoris (AP) and control groups (electron microscopy, bottom photo). All micrographs are shown at a magnification of ×40,000. The scale bar corresponds to 100 nm. MDA, malondialdehyde. ^#^P<0.01 vs. the control in the presence of cupric ion.

The HDL_3_ from the AP group had weaker antioxidant activity (172% increase from the initial level) than the control group (198% increase) when the same amount of protein in HDL (1.5 mg/ml) was used as an antioxidant source (Fig. 2A). The extent of oxidation in the native state was compared by relative electrophoretic mobility on 0.7% agarose gel electrophoresis. The HDL_2_ from the AP group migrated faster than the control group without cupric ion treatment, indicating that HDL_2_ of the AP group was more oxidized in the native state (Fig. 2B). More highly oxidized HDL has a faster mobility due to a smaller particle size and an increase in charge. In particular, HDL_2_ from the AP group was 2-fold more susceptible to cupric ion-mediated oxidation, as shown in Fig. 3, indicating that the antioxidant potential was significantly decreased in the AP group. Specifically, electron microscopy revealed that HDL_2_ from the AP group had a smaller particle size than the control; HDL_2_ from the AP group was 18–20 nm in width and length, while HDL_2_ from the control group was 22–25 nm in width and length. These results suggest that more highly oxidized HDL has faster electromobility and reduced particle size.

### HDL-associated paraoxonase and PAF-AH

**Figure 4:**
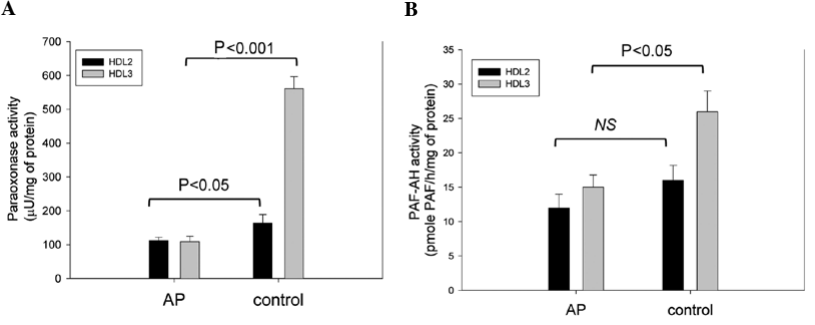
Activities of HDL-associated antioxidant enzymes, (A) paraoxonase and (B) platelet activating factor-acetylhydrolase (PAF-AH), between the angina pectoris (AP) and control groups. An equal amount of individual HDL_2_ and HDL_3_ were used as each enzyme source.

The HDL_2_-associated PON activity was lower in the AP group than in the control group (112±10 vs. 164±25 μU/mg of protein) (Fig. 4A). Moreover, the AP group had a 3-fold lower HDL_3_-associated PON activity than the control group (109±16 vs. 561±36 μU/mg of protein, respectively).

Although there was no significant difference in the HDL_2_ fraction used as the PAF-AH source, the activity was significantly lower in the AP group when the HDL_3_ fraction was used (Fig. 4). HDL_3_ from the AP group showed 40% less activity than the control group (15±2 and 26±3 pmole PAF/h/mg of protein for the AP and control groups, respectively).

### LDL from AP patient was more oxidized

**Figure 5:**
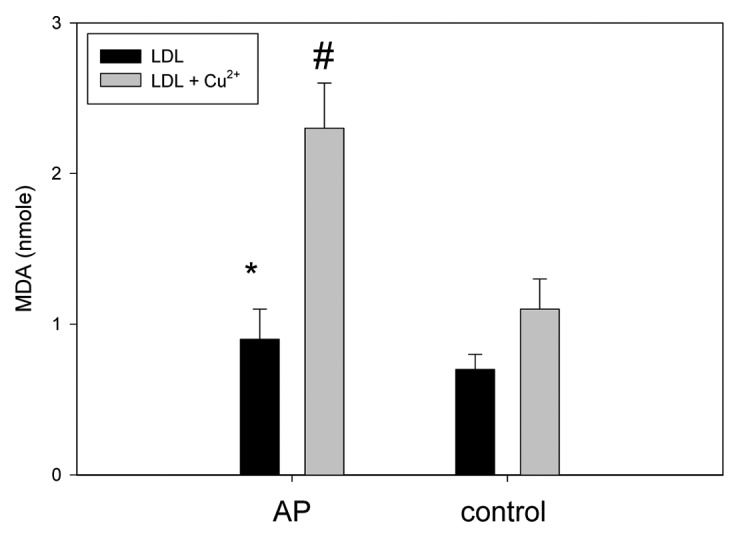
Oxidized extent of LDL with or without cupric ion treatment between the angina pectoris (AP) and control groups. ^*^P<0.05 and ^#^P<0.01 vs. the control.

The LDL from the AP patient had ~1.8-fold higher levels of MDA than the control without cupric ion treatment, indicating a greater extent of oxidation of LDL in the AP group in the native state (Fig. 5). Under treatment with cupric ion (final 10 μM), the LDL from the AP and control group showed 2.3 and 1.1 nmole of MDA, respectively, suggesting that the AP group LDL was more sensitive to cupric-ion mediated oxidation.

## Discussion

In addition to a change in serum lipid parameters, lipid and protein compositions in lipoproteins have emerged as a parameter which is associated with the progress of metabolic diseases, such as metabolic syndrome (13,14) and CHD (15). In fact, structural and functional changes in HDL are more dramatic in the acute phase, such as viral infections (25) and after cardiac surgery (26).

Although the AP group had similar levels of TC and LDL-C, the AP group had a lower ratio of HDL-C/TC. While the TC content was lower in HDL_2_ from the AP group, the TG content was significantly elevated. An increase in TG in the serum is a potent inflammatory factor and is associated with the incidence of CAD (27). Accumulation of serum TG in HDL has been correlated with the incidence of cardiovascular disease (4,10). TG-enriched lipoprotein is more inflammatory in vascular events (28). An elevated TG/HDL-C ratio is associated with increased insulin resistance and cardiovascular events (29). The current report suggests that the serum TG was more highly accumulated in the LDL and HDL_2_ fractions, rather than in the VLDL fraction, which is similar to the results of a previous report involving a male MI patient (10) that showed a strong and consistent association of hypertriglyceridemia with enriched LDL fraction. Recently, TG/HDL-C was shown to be a strong independent predictor of mortality in women with an ischemia syndrome (4). Several reports have suggested that an increased TG level is associated with elevation of apoC-III in lipoproteins; apoC-III in VLDL and LDL is linked with CHD and senescence (11,30). In the current study, TG in HDL_2_ and LDL, and CETP activity were elevated in AP patients, suggesting that apoC-III in HDL is also a risk factor for coronary events in female AP patients (Table I and Fig. 1).

It is known that serum CETP is an atherogenic factor. CETP promotes the transfer of CE from HDL to VLDL and LDL in exchange for TG, which moves in the opposite direction. The exchange of CE and TG between lipoproteins is linked to elevated levels of TG-enriched lipoprotein, which is pro-inflammatory and pro-atherogenic (31). CETP is an independent risk factor for CHD and metabolic syndrome (32). In addition, we recently reported that the metabolic syndrome in male patients is characterized by a 38% higher serum cholesteryl ester transfer protein (CETP) activity than the control group (10). The increase in TG is also associated with elevated level of apoC-III in the serum and lipoproteins in male MI patients (10). Furthermore, CETP activity is not decreased when apoC-III-enriched HDL is used as a CETP source (20). The current report showed that the AP group had an elevated level of apoC-III in HDL_3_.

With the alteration in the lipid content in HDL, many reports have suggested that HDL particle size is associated with cardiovascular events (9). Zeller *et al* (33) proposed that the smaller particle size of HDL is associated with young age in patients with acute MI. In addition, Arsenault *et al* (34) reported that a decreased HDL particle size is associated with an adverse cardiometabolic risk profile. They also proposed that a small HDL particle size was associated with an increased CHD risk. Interestingly, the HDL particle size was inversely related to CETP activity, serum TG concentration, body mass index, and C-reactive protein.

One of the beneficial virtues of HDL is exerting antioxidant activity. The increase in oxidation susceptibility in the AP group might be linked to alteration of lipid and protein composition in HDL. In the AP group, HDL_2_-TC was ~40% lower than the control, while HDL_2_-TG was elevated by 60%. Moreover, LCAT activity in HDL_2_ and HDL_3_ was 40 and 72% lower in the AP group, respectively, compared to the control. Using immunodetection techniques, LCAT expression was undetectable in the HDL_3_ fraction of the AP group with the exception of one patient, while the LCAT band was detected in the control (Fig. 1). The decrease in LCAT activity and expression may contribute to the loss of antioxidant activity and oxidation sensitivity.

In addition, human serum PON (EC 3.1.1.2) is an HDL-associated calcium-dependent enzyme, and has strong antioxidant activity. It catalyzes the hydrolysis of oxidized fatty acids from phospholipids and prevents the accumulation of oxidized lipids in lipoproteins, particularly LDL (23). PON activity and -SH levels have been shown to be lower in CAD patients (35), which suggests that reduced PON activity may contribute to the severity of CAD. PAF-AH (EC 3.1.1.47) is also involved in the antioxidant and anti-inflammatory functions associated with the surfaces of HDL (36), and is a Ca^2+^-independent enzyme belonging to group 7 of the PLA_2_ family (37). PAF-AH degrades oxidized phospholipids and platelet activating factor, which is a pro-inflammatory factor. Thus, PAF-AH may function as a profoundly anti-atherogenic enzyme. These three enzymes were coincidentally lowered in the HDL fraction of the AP group, which is in good agreement with decreased antioxidant activity.

In conclusion, the current results strongly support the interrelationship between CETP activity, the serum TG level and its distribution, apoC-III expression, and that the change in HDL particle size and antioxidant ability are intimately correlated, especially in the onset of the female with AP.

## Figures and Tables

**Table I:** Lipid and protein composition of lipoproteins from patients.

	Angina pectoris (n=22)			Control (n=20)		
		
	TC (mg/dl)	TG (mg/dl)	TP (mg/ml)	TC (mg/dl)	TG (mg/dl)	TP (mg/dl)
VLDL	120±64	203±140	2.8±0.1	130±31	243±46	2.8±0.1
LDL	1095±231	256±65^a^	6.6±0.2	909±177	165±15	5.5±1.3
HDL_2_	46±14^a^	82±24^a^	2.0±0.1	75±14	52±28	1.8±0.1
HDL_3_	64±16	16±7	3.6±0.5	58±22	24±22	3.8±0.1

a P<0.05 vs. control. VLDL, very low-density lipoprotein; LDL, low-density lipoprotein; HDL, high-density lipoprotein; TC, total cholesterol; TG, triacylglycerol; TP, total protein.

**Table II:** LCAT and CETP activities in lipoprotein fractions.

	Angina pectoris (n=22)	Control (n=20)
CETP activity^a^		
VLDL	2.6±0.6	3.1±0.1
LDL	5.5±0.3^c^	2.0±1.0
HDL_2_	17±2.1^c^	10±3.6
HDL_3_	35.4±6.6^c^	26.8±2.1
LCAT activity^b^		
HDL_2_	1.2±0.7	2.0±1.5
HDL_3_	3.5±1.3^c^	12.3±2.1

a CETP activity is expressed as % CE transfer/4 h.

b LCAT activity is expressed as % CE conversion/h/100 μg of protein in HDL.

c P<0.05 vs. control. LCAT, lecithin:cholesterol acyltransferase; CETP, cholesteryl ester transfer protein; VLDL, very low-density lipoprotein; LDL, low-density lipoprotein; HDL, high-density lipoprotein.

## References

[1] Rosamond W Flegal K Furie K Go A Greenlund K Haase N Hailpern SM Ho M Howard V Kissela B Kittner S Lloyd-Jones D McDermott M Meigs J Moy C Nichol G O’Donnell C Roger V Sorlie P Steinberger J Thom T Wilson M Hong Y American Heart Association Statistics Committee and Stroke Statistics Subcommittee Heart disease and stroke statistics-2008 update: a report from the American Heart Association Statistics Committee and Stroke Statistics Subcommittee Circulation 117 e25 e146 2008 18086926 10.1161/CIRCULATIONAHA.107.187998

[2] Cannon C Braunwald E Unstable angina and non-ST elevation myocardial infarction In: Harrison’s Principles of Internal Medicine Kasper DL Braunwald E Fauci AS Hauser SL Longo DL Jameson JL 16th edition McGraw-Hill New York 1444 1448 2005

[3] Jawad E Arora R Chronic stable angina pectoris Dis Mon 54 671 689 2008 18725007 10.1016/j.disamonth.2008.06.009

[4] Bittner V Johnson BD Zineh I Rogers WJ Vido D Marroquin OC Bairey-Merz CN Sopko G The triglyceride/high-density lipoprotein cholesterol ratio predicts all-cause mortality in women with suspected myocardial ischemia: a report from the Women’s Ischemia Syndrome Evaluation (WISE) Am Heart J 157 548 555 2009 19249427 10.1016/j.ahj.2008.11.014 PMC2677623

[5] Thygesen K Alpert JS White HD Joint ESC/ACCF/AHA/WHF Task Force for the Redefinition of Myocardial Infarction Jaffe AS Apple FS Galvani M Katus HA Newby LK Ravkilde J Chaitman B Clemmensen PM Dellborg M Hod H Porela P Underwood R Bax JJ Beller GA Bonow R Van der Wall EE Bassand JP Wijns W Ferguson TB Steg PG Uretsky BF Williams DO Armstrong PW Antman EM Fox KA Hamm CW Ohman EM Simoons ML Poole-Wilson PA Gurfinkel EP Lopez-Sendon JL Pais P Mendis S Zhu JR Wallentin LC Fernández-Avilés F Fox KM Parkhomenko AN Priori SG Tendera M Voipio-Pulkki LM Vahanian A Camm AJ De Caterina R Dean V Dickstein K Filippatos G Funck-Brentano C Hellemans I Kristensen SD McGregor K Sechtem U Silber S Tendera M Widimsky P Zamorano JL Morais J Brener S Harrington R Morrow D Lim M Martinez-Rios MA Steinhubl S Levine GN Gibler WB Goff D Tubaro M Dudek D Al-Attar N Universal definition of myocardial infarction Circulation 116 2634 2653 2007 17951284 10.1161/CIRCULATIONAHA.107.187397

[6] Walldius G Jungner I Is there a better marker of cardiovascular risk than LDL cholesterol? Apolipoproteins B and A-I-new risk factors and targets for therapy Nutr Metab Cardiovasc Dis 17 565 571 2007 17631989 10.1016/j.numecd.2007.02.010

[7] Tsimikas S Willerson JT Ridker PM C-Reactive protein and other emerging blood biomarkers to optimize risk stratification of vulnerable patients J Am Coll Cardiol 47 C19 C31 2006 16631506 10.1016/j.jacc.2005.10.066

[8] Huang XS Zhao SP Zhang Q Bai L Hu M Elevated plasma apolipoprotein AV in acute coronary syndrome is positively correlated with triglyceride and C-reactive protein Chin Med J (Engl) 122 1408 1412 2009 19567162

[9] Tashiro J Miyazaki O Nakamura Y Miyazaki A Fukamachi I Bujo H Saito Y Plasma pre beta1-HDL level is elevated in unstable angina pectoris Atherosclerosis 204 595 600 2009 19054517 10.1016/j.atherosclerosis.2008.10.015

[10] Cho KH Shin DG Baek SH Kim JR Myocardial infarction patients showed altered lipoprotein properties and functions when compared with stable angina pectoris patients Exp Mol Med 41 67 76 2009 19287187 10.3858/emm.2009.41.2.009 PMC2679332

[11] Lee SJ Campos H Moye LA Sacks FM LDL containing apolipoprotein CIII is an independent risk factor for coronary events in diabetic patients Arterioscler Thromb Vasc Biol 23 853 858 2003 12637336 10.1161/01.ATV.0000066131.01313.EB

[12] Hartford M Wiklund O Mattsson Hultén L Persson A Karlsson T Herlitz J Caidahl K C-reactive protein, interleukin-6, secretory phospholipase A group IIA and intercellular adhesion molecule-1 in the prediction of late outcome events after acute coronary syndromes J Intern Med 262 526 536 2007 17908161 10.1111/j.1365-2796.2007.01862.x

[13] Park KH Shin DG Kim JR Cho KH The functional and compositional properties of lipoproteins are altered in patients with metabolic syndrome with increased cholesteryl ester transfer protein activity Int J Mol Med 25 129 136 2010 19956911

[14] McLaughlin T Abbasi F Cheal K Chu J Lamendola C Reaven G Use of metabolic markers to identify overweight individuals who are insulin resistant Ann Intern Med 139 802 809 2003 14623617 10.7326/0003-4819-139-10-200311180-00007

[15] Genest JJ Jr Martin-Munley SS McNamara JR Ordovas JM Jenner J Myers RH Silberman SR Wilson PW Salem DN Schaefer EJ Familial lipoprotein disorders in patients with premature coronary artery disease Circulation 85 2025 2033 1992 1534286 10.1161/01.cir.85.6.2025

[16] Havel RJ Eder HA Bragdon JH The distribution and chemical composition of ultracentrifugally separated lipoproteins in human serum J Clin Invest 34 1345 1353 1955 13252080 10.1172/JCI103182 PMC438705

[17] Markwell MA Haas SM Bieber LL Tolbert NE A modification of the Lowry procedure to simplify protein determination in membrane and lipoprotein samples Anal Biochem 87 206 210 1978 98070 10.1016/0003-2697(78)90586-9

[18] Blois MS Antioxidant determinations by the use of a stable free radical Nature 181 1199 1200 1958

[19] Benzie IF Strain JJ The ferric reducing ability of plasma (FRAP) as a measure of antioxidant power: the FRAP assay Anal Biochem 239 70 76 1996 8660627 10.1006/abio.1996.0292

[20] Cho KH Synthesis of reconstituted high-density lipoprotein (rHDL) containing apoA-I and apoC-III: the functional role of apoC-III in rHDL Mol Cells 27 291 297 2009 19326075 10.1007/s10059-009-0037-8

[21] Han JM Jeong TS Lee WS Choi I Cho KH Structural and functional properties of V156K and A158E mutants of apolipoprotein A-I in the lipid-free and lipid-bound states J Lipid Res 46 589 596 2005 15716588 10.1194/jlr.M400468-JLR200

[22] Cho KH Lee JY Choi MS Cho JM Lim JS Park YB A peptide from hog plasma that inhibits human cholesteryl ester transfer protein Biochim Biophys Acta 1391 133 144 1998 9554982 10.1016/s0005-2760(97)00197-5

[23] Eckerson HW Wyte CM La Du BN The human serum paraoxonase/arylesterase polymorphism Am J Hum Genet 35 1126 1138 1983 6316781 PMC1685985

[24] Boyd HF Fell SC Flynn ST Hickey DM Ife RJ Leach CA Macphee CH Milliner KJ Moores KE Pinto IL Porter RA Rawlings DA Smith SA Stansfield IG Tew DG Theobald CJ Whittaker CM N-1 substituted pyrimidin-4-ones: novel, orally active inhibitors of lipoprotein-associated phospholipase A2 Bioorg Med Chem Lett 10 2557 2561 2000 11086729 10.1016/s0960-894x(00)00510-2

[25] Cho KH Park SH Park JE Kim YO Choi I Kim JJ Kim JR The function, composition, and particle size of high-density lipoprotein were severely impaired in an oliguric phase of hemorrhagic fever with renal syndrome Clin Biochem 41 56 64 2008 17996200 10.1016/j.clinbiochem.2007.10.007

[26] Jahangiri A de Beer MC Noffsinger V Tannock LR Ramaiah C Webb NR van der Westhuyzen DR de Beer FC HDL remodeling during the acute phase response Arterioscler Thromb Vasc Biol 29 261 267 2009 19008529 10.1161/ATVBAHA.108.178681 PMC2760005

[27] McBride PE Triglycerides and risk for coronary heart disease J Am Med Assoc 298 336 338 2007 10.1001/jama.298.3.336 17635897

[28] Libby P Fat fuels the flame triglyceride-rich lipoproteins and arterial inflammation Circulation 100 299 301 2007 10.1161/01.RES.0000259393.89870.58 17307968

[29] Ostfeld R Mookherjee D Spinelli M Holtzman D Shoyeb A Schaefer M Doddamani S Spevack D Du Y A triglyceride/high-density lipoprotein ratio > or = 3.5 is associated with an increased burden of coronary artery disease on cardiac catheterization J Cardiometab Syndr 1 13 15 2006 17675905 10.1111/j.0197-3118.2006.05323.x

[30] Park KH Shin DG Kim JR Cho KH Senescence-related truncation and multimerization of apolipoprotein A-I in high-density lipoprotein with an elevated level of advanced glycated end products and cholesteryl ester transfer activity J Gerontol A Biol Sci Med Sci 65 600 610 2010 20421239 10.1093/gerona/glq034

[31] Chapman MJ Goff WL Guerin M Kontush A Cholesteryl ester transfer protein: at the heart of the action of lipid-modulating therapy with statins, fibrates, niacin, and cholesteryl ester transfer protein inhibitors Eur Heart J 31 149 164 2010 19825813 10.1093/eurheartj/ehp399 PMC2806550

[32] Chapman MJ Therapeutic elevation of HDL-cholesterol to prevent atherosclerosis and coronary heart disease Pharmacol Ther 111 893 908 2006 16574234 10.1016/j.pharmthera.2006.02.003

[33] Zeller M Masson D Farnier M Lorgis L Deckert V Pais de Barros JP Desrumaux C Sicard P Grober J Blache D Gambert P Rochette L Cottin Y Lagrost L High serum cholesteryl ester transfer rates and small high-density lipoproteins are associated with young age in patients with acute myocardial infarction J Am Coll Cardiol 50 1948 1955 2007 17996559 10.1016/j.jacc.2007.06.052

[34] Arsenault BJ Lemieux I Després JP Gagnon P Wareham NJ Stroes ES Kastelein JJ Khaw KT Boekholdt SM HDL particle size and the risk of coronary heart disease in apparently healthy men and women: the EPIC-Norfolk prospective population study Atherosclerosis 206 276 281 2009 19268944 10.1016/j.atherosclerosis.2009.01.044

[35] Gur M Aslan M Yildiz A Demirbag R Yilmaz R Selek S Erel O Ozdogru I Paraoxonase and arylesterase activities in coronary artery disease Eur J Clin Invest 36 779 787 2006 17032345 10.1111/j.1365-2362.2006.01727.x

[36] Karasawa K Clinical aspects of plasma platelet-activating factor-acetylhydrolase Biochim Biophys Acta 1761 1359 1372 2006 17049457 10.1016/j.bbalip.2006.06.017

[37] Six DA Dennis EA The expanding superfamily of phospholipase A(2) enzymes: classification and characterization Biochim Biophys Acta 1488 1 19 2000 11080672 10.1016/s1388-1981(00)00105-0

